# The Ukrainian version of the Perceived Injustice Questionnaire: A psychometric evaluation

**DOI:** 10.3389/fpsyt.2024.1446724

**Published:** 2024-12-02

**Authors:** Anna Weigelt, Jan Ilhan Kizilhan

**Affiliations:** ^1^ Institute for Transcultural Health Science, Baden-Wuerttemberg Cooperative State University, Stuttgart, Germany; ^2^ Institute for Psychotherapy and Psychotraumatology, University of Duhok, Duhok, Iraq

**Keywords:** injustice, trauma, war, PTSD, psychotherapy, trauma therapy

## Abstract

**Introduction:**

Perceived injustice is increasingly recognized as a key factor influencing mental health in war survivors. This cross-sectional study aimed to evaluate the psychometric properties of the Ukrainian translation of the Perceived Injustice Questionnaire (PIQ) among individuals directly exposed to the war in Ukraine.

**Methods:**

170 war-exposed Ukrainians completed the Ukrainian PIQ and measures of post-traumatic tress disorder (PCL-5), depression (PHQ-9), generalized anxiety disorder (GAD-7), and potentially traumatic life events (LEC-5). Internal consistency, factor structure, and criterion validity were assessed.

**Results:**

While the factor structure of the English version could not be reproduced, indicating an issue with factor validity in the Ukrainian version, the Ukrainian PIQ demonstrated strong correlations with post-traumatic stress disorder (PTSD) symptoms (r = .71, *p* <.01), moderate associations with depression (r = .62, *p* <.01) and generalized anxiety disorder (r = .61, *p* <.01), and a weaker link with potentially traumatic life events (PTLEs, r = .35, *p* <.01). It significantly predicted PTSD, depression, and generalized anxiety disorder (GAD) symptoms beyond PTLE exposure, explaining 33% of variance in depression, 31% in GAD, and 45% in PTSD These findings provide evidence supporting the construct validity of the PIQ in terms of its relationships with mental health outcomes. The Ukrainian PIQ also demonstrated excellent overall reliability (α = .90), with factor reliabilities ranging from α = .74 to α = .81.

**Conclusions:**

The Ukrainian version of the PIQ demonstrates promising psychometric properties and emerges as a highly significant correlate of mental health outcomes. This underscores its potential utility in clinical practice for assessing the treatment needs of Ukrainians affected by the consequences of war. Moreover, the findings highlight the importance of developing therapy modules specifically tailored to address perceived injustice. Further research is warranted to investigate the cross-cultural adaptability and comparability of the PIQ.

## 1 Introduction

After the full-scale invasion of Ukraine on 24 February 2022, a catastrophic health and humanitarian crisis of unforeseeable proportions was unleashed. In addition to the widespread destruction of critical infrastructure, which left millions without access to water and electricity for extended periods (Luxmoore, 2022), numerous documented human rights violations occurred (1, 2).

While the tangible consequences of armed conflicts — shattered buildings, displaced masses, human losses — are evident, the impact on mental well-being remains less visible yet equally critical. Extensive research has demonstrated a direct association between exposure to life-threatening traumatic events and the increased risk of mental illnesses (3). Additionally, there is substantial evidence that the prevalence of mental disorders tends to rise in the aftermath of war exposure and displacement, with PTSD, depression, and anxiety disorders being the most commonly observed conditions within this context (4, 5).

Ukraine carried a significant burden of mental illnesses even before the invasion began. Globally, approximately one in four individuals is expected to experience a mental disorder during their lifetime (6). In Ukraine, the lifetime prevalence of mental illnesses stood at 31.6% (7). In 2014, the country’s most common mental disorders included depressive disorders with a prevalence of 6.31%, anxiety disorders with a prevalence of 3.18%, and alcohol use disorders with a prevalence of 2.26% (8). In the years thereafter, higher rates of mental illnesses were observed, particularly in Eastern Ukraine and among internally displaced individuals due to the Eastern Ukrainian conflict. Prevalence rates for displaced Ukrainians ranged from 18-32% for PTSD, 22-32% for depression, and 17-56% for anxiety disorders (9–11). Recent studies have reported even higher prevalence rates, such as PTSD ranging from 25-74% (12–14), depression symptoms between 42-46%, and anxiety symptoms between 35-54% among the general Ukrainian (15–17). The most vulnerable groups are internally displaced Ukrainians and those who sought refuge in other countries (12, 18). Given the significant burden of mental illnesses in Ukraine, there is an urgent call for research aimed at understanding the morbidity and risk factors associated with mental health disorders in this vulnerable population.

A promising concept in this regard is the consideration of perceived injustice. How individuals perceive and respond to acts of injustice not only shapes their actions but also leaves lasting imprints on their physical and mental well-being. In the realm of pain and rehabilitation studies, a growing body of research highlights the significant influence of perceived injustice on the perception of pain, the rehabilitation process, and the development of post-traumatic stress symptoms as well as depression (19–22). While the field of pain research is currently at the forefront of addressing these issues, the exploration of perceived injustice is not a recent endeavor. Organizational psychology, for example, laid the initial groundwork by establishing the link between perceived injustice in workplace settings and stress-related symptoms (23, 24). Forensic studies within the penal system (25) and social psychology investigations into gender-specific perceptions of injustice within families (26) have also contributed to the understanding of this issue. Yet, the complex interplay between perceived injustice, traumatic experiences, and mental health remains a subject of an ongoing inquiry. Research following violent conflicts in places such as Rwanda and Timor-Leste indicates a possible link between traumatic events, post-traumatic stress disorder (PTSD), and perceived injustice (27, 28). However, not all studies in post-conflict regions have managed to empirically confirm this connection (29). To comprehensively grasp the impact of perceived injustice after war and flight on mental health, researchers have recognized the crucial need for rigorous and validated assessment tools. Neumann et al. (30) have developed a transcultural instrument for assessing perceived injustice following war, conflict, and displacement – The Perceived Injustice Questionnaire (PIQ). The initial validation of the PIQ took place in Northern Iraq, demonstrating its potential to provide valuable insights into the nuanced landscape of post-conflict injustice perception.

In line with the goal of creating a transculturally applicable instrument, the PIQ underwent translation into Ukrainian as part of this study, accompanied by a comprehensive analysis of its psychometric properties. This analysis encompasses considerations of construct validity, reliability, criterion validity, and incremental validity. The primary purpose of this research is to ascertain whether the Ukrainian version of the PIQ effectively measures perceived injustice while maintaining robust validity and reliability. Further, the study aims to assess its potential utility in clinical settings, where targeting perceived injustice could be beneficial in addressing symptoms related to depression, PTSD, and anxiety. In accordance with this objective, following hypotheses were examined:

1. The Ukrainian version of the PIQ closely aligns with the data structure of the original English version.
2. The overall test of the Ukrainian version as well as the subscales demonstrate good reliability.
3. Exposure to war-related potentially traumatic life events positively correlate with perceived injustice.
4. Perceived injustice positively correlates with elevated symptoms of depression, generalized anxiety disorder and of PTSD.
5. The level of perceived injustice accounts for symptoms of depression, generalized anxiety disorder and of PTSD, surpassing the impact of potentially traumatic events.

By addressing these inquiries, the study contributes to the expanding field of transcultural psychotherapy research, providing trauma therapists with a valuable tool for conducting nuanced problem assessments and guiding their psychotherapeutic interventions.

## 2 Materials and methods

### 2.1 Translation process

The translation of the PIQ adhered to contemporary guidelines for translating questionnaires in the medical field (31). Two native Ukrainian speakers undertook the initial translation from English to Ukrainian, discussing discrepancies in their translations. Subsequently, the Ukrainian version was retranslated into English by a third party. Lastly, all translators collaboratively reviewed disparities between the original version and the English back translation.

### 2.2 Sample

Ukrainians directly affected by the war in Ukraine, including those who had fled to different countries, those who were internally displaced within Ukraine, and those who had remained in their original communities, were recruited with the assistance of activists, trauma researchers, therapists, and refugee psychosocial care centers. The recruitment took place between January and June 2023, using online methods like social media posts and brochures with a QR link to the questionnaire. The study was completed by 175 of the 292 participants. Minors and those who did not provide consent to participate were excluded, resulting in a final sample of 170 participants for data analysis. With a subject-to-variable ratio of 9.44, the sample size of the current study exceeded the minimum requirements for factor analysis (32, 33). With a desired power of.80, a minimum sample size of 128 was sufficient to assess PIQ’s predictive power beyond LEC-5 using ANOVA (34). Our sample of 170 participants comfortably exceeded this threshold.

### 2.3 Measures

#### 2.3.1 Perceived injustice questionnaire

The PIQ (30) is a 19-item self-report questionnaire developed to assess how individuals perceive injustice in their experiences of conflict and war. It encompasses four scales measuring Emotional and Cognitive Consequences (EEC), Injustice Perception (IP), Injustice Experiences (IE), and Revenge and Forgiveness (RF), as well as five additional items, which are not assigned to any of the scales. All items show an acceptable to satisfactory reliability and are answered on a 5-point Likert rating scale ranging from 1 (“strongly agree”) to 5 (“strongly disagree”) **(Appendix A)**. To determine the level of perceived injustice, the mean value for each subscale is computed, summed together, and divided by the number of the scales. The additional items are also considered and form an independent scale. The Ukrainian version of the PIQ **(Appendix B)** also employs a 5-point Likert scale, with the difference that 1 stands for “strongly disagree” and 5 for “strongly agree”. This adjustment was implemented based on findings that suggested that arranging response options in a descending order can potentially result in inflated scores (35, 36).

#### 2.3.2 Life events checklist for DSM-5

The LEC-5 (37) is a 17-item self-report questionnaire designed to assess potentially traumatic life events (PTLE) that may have occurred over the respondent’s lifetime. Participants are presented with six levels of exposure options, ranging from “happened to me” to “witnessed,” “learned about it,” “part of my job,” “not sure,” and “does not apply.” While the psychometric characteristics of the LEC-5 have not been extensively studied, there is evidence of fair-to-good 3-month stability and good construct validity (38, 39). The slightly different LEC for DSM-IV version demonstrates strong convergence with other trauma measures (40). There have been no investigations on the psychometric features of the Ukrainian language version of the LEC-5, which is available online as part of an open science project (41). In this study, the focus was specifically on experiences related to the war in Ukraine, with the intention to exclude non-war-related events. Therefore, only 14 out of 17 items from the LEC-5 were utilized, omitting the following items: “Natural disaster (for example, flood, hurricane, tornado, earthquake)”, “Serious accident at work, home, or during recreational activity”, and “Any other very stressful event or experience”. Furthermore, this study focused solely on potentially traumatic events that occurred during the war in Ukraine, rather than over the lifespan.

#### 2.3.3 Patient health questionnaire-9

The PHQ-9 (42) is a self-report questionnaire developed to assess the severity of depression symptoms in individuals. It comprises nine items, each corresponding to the nine diagnostic criteria for major depressive disorder as outlined in the Diagnostic and Statistical Manual of Mental Disorders (DSM-5). Participants are asked to rate the frequency of experiencing each symptom over the previous two weeks, with response options ranging from 0 (“not at all”) to 3 (“nearly every day”). The PHQ-9 demonstrates a high sensitivity and specificity of 88% in detecting major depressive disorder, along with high internal consistency (α = .89), excellent test-retest-reliability (ICC = .84), good construct validity, and substantial convergence with other depression measures (42–44). Recent studies employing the Ukrainian version of the PHQ-9 have reported very good internal reliability (α = .90), test-retest reliability (ICC = .84), and good construct validity (11, 45). In line with previous research, the PHQ-9 exhibited good internal reliability (α = .84) in this study.

#### 2.3.4 Posttraumatic stress disorder checklist for DSM-5

The PCL-5 (46) is a self-report questionnaire designed to assess the presence and severity of PTSD symptoms in individuals. It consists of 20 items that align with the diagnostic criteria for PTSD in the DSM-5. Participants are asked to rate the severity of each symptom during the previous month, using response options ranging from 0 (“not at all”) to 4 (“extremely”). The PCL-5 exhibits high internal consistency (α = .94), test-retest reliability (ICC = .82), good convergent, discriminant, and it effectively discriminates between individuals with and without PTSD (46, 47). Studies employing the Ukrainian version of the PCL-5 reported very high internal reliability (α = .95 and.96), good test-retest reliability (ICC = .83) as well as strong construct validity (11, 48). In this study, the reliability of the Ukrainian PCL-5 was found to be excellent, with an alpha coefficient of α = .93.

#### 2.3.5 Generalized anxiety disorder 7-item scale

The GAD-7 (49) is a self-report questionnaire developed to assess the severity of generalized anxiety disorder symptoms in individuals. It comprises seven items that align with the DSM-5 diagnostic criteria for generalized anxiety disorder. Participants are asked to rate the frequency of experiencing each symptom over the past two weeks, using response options ranging from 0 (“not at all”) to 3 (“nearly every day”). The GAD-7 demonstrates high internal consistency (α = .92), test-retest reliability (ICC = .83), good convergent validity, and effective discrimination between individuals with generalized anxiety disorder and those without (49, 50). Studies utilizing the Ukrainian version of the GAD-7 have reported very high internal reliability (α = .93 and.92), test-retest reliability (ICC = .89), and strong construct validity (11, 45). In line with previous studies, the GAD-7 exhibited an internal reliability of α = 0.90.

### 2.4 Procedure

The survey was conducted on the online survey platform LimeSurvey (51). Data were collected at a single time point, reflecting the study’s cross-sectional design. It adhered to ethical guidelines as outlined by the German Psychological Society (52). Participants were provided with clear information about the study’s objectives, the data collection process, and potential risks before giving their informed consent to participate. Participation was entirely voluntary, with no monetary compensation offered, ensuring participants’ autonomy. To address any potential psychological distress, a list of contact points was included at the end of the survey. Every effort was made to minimize the duration of the survey, which took 15 minutes to complete, and it was accessible on various devices, including laptops, mobile phones, and tablets.

### 2.5 Data analysis

The data were analyzed using the statistical software R (53). After data screening and z-standardization of all questionnaires, a confirmatory factor analysis (CFA) was conducted. The weighted least square mean and variance-adjusted (WLSMV) estimation method was used because it is particularly well-suited for non-normally distributed Likert-scale data (54). Due to an oversight in the translation process, one item from the IE scale was omitted, resulting in an incomplete factor identification. To ensure the identification of the IE factor, the factor loadings of the two remaining IE items were set to 1. To validate the robustness of this approach, a second factor identification method was implemented. In the subsequent run, the IE factor variance was constrained to 1, while the factor loadings were estimated. Fit indices following recommendations by Hu and Bentler (55, 56), Cho et al. (57), Iacobucci et al. (58), and DiStefano et al. (59) were evaluated (**Table 1**).

**Table 1:** Fit indices.

Fit index	Recommended cut-off value
Chi-square	*p* >.05 (58)
Chi-square/degree of freedom	≤ 3 (58)
CFI	≥.95 (56)
TLI	≥.95 (56)
SRMR	≤.08 (56)
RMSEA	≤.06 (56)
WRMR	≤ 1.0 (59)
GFI	≥.93 (57)

CFI, Comparative Fit Index; TLI, Tucker-Lewis index; SRMR, Standardized Root Mean Squared Residual; RMSEA, Root Mean Square Error of Approximation; WRMR, Weighted Root Mean Square Residual; GFI, Goodness of Fit Index.

To gain a more comprehensive understanding of the result of the CFA, an Exploratory Factor Analysis (EFA) using weighted least squares (WLS) was conducted. All available items were included in this analysis, including item EEC7, which was introduced after the validation of the original scale by its authors and has since become an integral part of the questionnaire. To determine the number of components to retain for rotation, a combination of techniques recommended by Velicer et al. (60) was employed, including Parallel analysis (61) and Minimum Average Partial (MAP) test (62), with the Screen test used as a supplementary method. Additionally, the Bayesian Information Criterion (BIC) and the Sample Size Adjusted BIC were examined (63). Since Neumann et al. (30) did not provide theoretical justification for applying a principal component analysis (PCA), which assumes an orthogonal factor structure, a provision was made to allow for correlated factors by using oblique Promax rotation if Varimax rotation failed to produce a clear solution or if correlations between factors exceeded a threshold of 0.32 (64). To ensure the robustness of the results in terms of the rotation method, a comparison was made with an Oblimin solution (65). Lastly, a hierarchical g-factor model was examined to rigorously test the possibility that the model might be underpinned by a general factor. Following the recommendations by Watkins (66), the evaluation of model fit included the use of the Root Mean Squared Residual (RMSR), with a threshold set at ≤.08, as suggested by Brown (67). Additionally, the BIC was employed, favoring models with the lowest BIC values. Furthermore, the differences in RMSEA values were scrutinized, with a difference of ≥.015 considered to be statistically significant, aligning with the criteria outlined by Finch (68). Loadings exceeding.70 were classified as excellent, while those at.63 were considered as very good,.55 as good, and.45 as fair (69). The threshold for factor salience was set at.32 (70), aiming for an approximately simple factor structure (71). To ensure greater transparency and reproducibility, it was opted to consider and report the rotated eigenvalues (SS loadings) of the factors within the analysis (72).

To assess reliability, Cronbach’s alpha was calculated for the entire test and each scale. For clinical purposes, coefficients in the.90s were deemed excellent and adequate, those in the.80s were considered good and sufficient, and those in the.70s were interpreted as suitable for research, but not for individual diagnostics (73, 74).

To assess the criterion validity of the PIQ, a trauma sum score was derived from the nominally scaled LEC-5 responses, where “happened to me” was assigned 3 points, “witnessed” received 2 points, “learned about it” got 1 point, and “does not apply” was assigned 0 points. The relationships between PIQ, LEC-5, PCL-5, PHQ-9, and GAD-7 were assessed using Pearson correlation analysis (75). To determine whether the PIQ can predict symptoms of depression, PTSD, and anxiety disorders beyond exposure to potentially traumatic events, hierarchical multiple regression analyses were conducted. Following recommendations by Dancey and Reidy (76), Pearson’s correlation coefficients between.1 and.3 were interpreted as weak, between.4 and.6 as moderate, and between.7 and.9 as strong.

## 3 Results

### 3.1 Sample demographics

As sociodemographic information, age, gender, highest level of education, and flight experience were assessed. Demographic characteristics, as detailed in **Table 2**, indicated that participants ranged in age from 18 to 78 years (*M* = 35, *SD* = 12.84). The sample included 76.47% females and 22.94% males, while 0.59% of the participants identified as diverse. Educational backgrounds varied, with 73.53% holding a university degree. 11.18% graduated from a technical college, 14.71% completed high school, and 0.59% indicated that their highest level of education was secondary school. Furthermore, 67.65% reported fleeing abroad due to the war, 17.06% were internally displaced, and 15.30% had no prior flight experience.

**Table 2:** Sample characteristics.

Age	Min	Max	*M*	*SD*
	18	78	35.00	12.84
	N	% of Total sample		
Gender				
Female	130	76.47		
Male	39	22.94		
Diverse	1	0.59		
Highest level of education				
Secondary school	1	0.59		
High school	25	14.71		
Technical	19	11.18		
University	125	73.53		
Flight experience				
None	26	15.30		
Within Ukraine	29	17.06		
Abroad	115	67.65		

Min, Minimum; Max, Maximum; *M*, Mean; *SD*, Standard deviation; N, Sample size.

### 3.2 Data screening regarding PIQ-items

While the data exhibited no missing values, Mardia’s test indicated a non-normal multivariate distribution, as evidenced by a multivariate skewness of 57.08 (p <.001) and multivariate kurtosis of 392.36 (p <.001). However, skewness (-0.53 to 0.75) and kurtosis (-1.14 to 0.26) values fell within acceptable ranges for further analyses. The examination of boxplots did not reveal any significant outliers. Analysis of multivariate outliers using the Mahalanobis distance initially identified five individuals with outlier values. A plausibility check confirmed that these values were not the result of input errors, leading to the inclusion of all 170 subjects in the analysis. Further exploration through correlation matrices and bivariate scatterplots indicated exclusively linear relationships between the variables. Eigenvalues of the covariance matrix ranged from 0.24 to 6.70, signaling the absence of singularity issues. Correlations in the correlation matrix ranged from.04 to.65, suggesting no concerns related to multicollinearity. The determinant of the correlation matrix was found to be 0.0006, providing further evidence against multicollinearity. The absence of negative eigenvalues in the covariance matrix and the lack of negative diagonal elements in the inverse correlation matrix confirmed the data’s positive definiteness. The Kaiser-Meyer-Olkin measure yielded a value of 0.89, and Bartlett’s test of sphericity was statistically significant (p <.001), supporting the suitability of the data for factor analysis. No negative correlations or inversely formulated items were detected within the 18 PIQ items, facilitating the continuation of z-standardization as planned.

### 3.3 Descriptive analysis.

**Table 3** provides a descriptive overview of the study variables. The mean score for the PIQ was 2.83 (*SD* = 0.61), ranging from a minimum of 1.24 to a maximum of 4.35. For the PHQ-9, the mean was 2.39 (*SD* = 0.67), with scores ranging from 1.00 to 3.77. Similarly, the PCL-5 had a mean of 2.67 (*SD* = 0.85), with a minimum of 1.00 and a maximum of 5.00. The GAD-7 mean was 2.36 (*SD* = 0.76), ranging from 1.00 to 4.00. Finally, the LEC-5 had a mean of 9.99 (*SD* = 6.34), with scores ranging from 0 to 31.

**Table 3:** Descriptive analysis of used questionnaires.

	*M*	*SD*	Minimum	Maximum
PIQ	2.83	0.61	1.24	4.35
PHQ-9	2.39	0.67	1.00	3.77
PCL-5	2.67	0.85	1.00	5.00
GAD-7	2.36	0.76	1.00	4.00
LEC-5	9.99	6.34	0.00	31.00

M, Mean; SD, Standard deviation; PIQ, Perceived Injustice Questionnaire; LEC-5, Life Events Checklist for DSM-5; PHQ-9, Patient Health Questionnaire-9; PCL-5, Posttraumatic Stress Disorder Checklist for DSM-5; GAD-7, Generalized Anxiety Disorder 7-item scale.

### 3.4 Item analysis of the PIQ

The item means of the PIQ-items ranged from *M* = 2.07 to *M* = 3.55, with standard deviations spanning from *SD* = 0.80 to *SD* = 1.31. Item difficulty varied from *P_i_* = 30.44 to *P_i_* = 63.82, with item discriminant power ranging from *r_it_* = .41 to *r_it_* = .73. Corrected item-total correlations revealed values between.33 and.69, signifying a robust level of internal consistency reliability. An analysis of Cronbach’s Alpha, with each item systematically removed, resulted in reliabilities spanning from.89 to.90, signifying excellent reliability.

### 3.5 Confirmatory factor analysis

The hypothesized four-factor model for the Ukrainian PIQ exhibited poor fit, indicating a deviation from the original English version (χ² = 1972.61, df = 136, *p* <.001). The fit indices supported this conclusion (χ²/df = 14.50, robust CFI = .30, robust TLI = .21, robust RMSEA = .28 (90% C.I.  [.27-.30]), SRMR = .28, WRMR = 2.60 and GFI = .63). Regression coefficients ranged from 0.78 to 1.31 for EEC, from 0.70 to 1.04 for IP, and from 0.82 to 1.48 for RF, further indicating model misspecification (**Table 4**). The model where the variance of the IE factor was constrained to 1 yielded no improvements in fit indices. These findings suggest the Ukrainian PIQ data structure deviates from the original English version, thus rejecting Hypothesis 1.

**Table 4:** Confirmatory factor analysis of 18 PIQ-items.

	Loadings	Std. Err.	z-value	P(>|z|)
Emotional and cognitive consequences				
EEC1: I am scared that I will experience injustice again in my life.	1.00			
EEC2: I feel unsafe.	.98	.13	7.68	.000
EEC3: I feel betrayed by humanity and cannot trust many people.	.84	.14	6.07	.000
EEC4: I feel left alone.	.93	.13	7.14	.000
EEC5: I feel a lot of anger, range or aggression.	.81	.14	5.96	.000
EEC6: I feel guilty and/or ashamed.	.64	.14	4.71	.000
EEC7: I feel humiliated.	1.06	.14	7.51	.000
Injustice experiences				
IE2: I feel that acts of injustice that I have had experienced affected me in a permanent way.	1.00			
IE3: Some of the experiences that I have had were a lot worse than what humans generally experience.	1.00			
Injustice perception				
IP1: I am mistreated more often than other people.	1.00			
IP2: My life is a lot harder than the lives of people around me.	1.09	.28	3.97	.000
IP3: I often get less than I deserve.	1.24	.32	3.97	.000
IP4: I am suffering because of someone else’s negligence.	1.29	.34	3.82	.000
IP5: It all seems so unfair.	.86	.25	3.52	.000
Revenge and forgiveness				
RF1: Nothing will ever make up for what I have gone through.	1.00			
RF2: I want to punish the person who has hurt me.	1.74	.35	4.96	.000
RF3: I do not want to forgive, I want revenge.	1.80	.37	4.83	.000
RF4: I cannot forgive the people who did not help me.	1.22	.27	4.60	.000

### 3.6 Explanatory factor analysis

Parallel analysis, Scree test, and the Sample Size Adjusted BIC results indicated a four-factorial model, while BIC pointed to two factors and Velicer’s minimum average partial (MAP) test to a one factor solution. Therefore, the interpretability and theoretical significance of models with four, two, and one factor was evaluated using the Varimax rotation method. Fit indices are presented in **Table 5**. While the four-factor solution demonstrated the best model fit, the Varimax rotation did not provide a clear result due to 10 cross-loading items. Consequently, EFA with Promax rotation was conducted.

**Table 5:** Model comparison of explanatory factor analysis with varimax rotation.

Model	RMSR	BIC	RMSEA
One-factor solution	.09	-325.40	.10
Two-factor solution	.07	-333.64	.09
Four-factor solution	.04	-333.45	.03

RMSR, Root Mean Squared Residual; BIC, Bayesian Information Criterion; RMSEA, Root Mean Square Error of Approximation.

Promax rotation produced comparable model fit indices to Varimax (RMSR = .04, BIC = -333.45, RMSEA = 0.03), with only one cross-loading item. Inter-factor correlations, ranging from.51 to.59, supported an oblique rotation. Given a good model fit, a clear structure, and relevant factor correlations, it was decided to accept the model with Promax rotation.

The results of the EFA with Promax rotation are summarized in **Table 6**. The rotated eigenvalue (SS loading) for Factor 1 was 2.38, explaining 13% of the variance, with factor loadings ranging from .51 to .64 and communalities between .31 and .57. One cross-loading item (EEC6) loaded on Factor 1 at.52 and on Factor 4 at -.34. Factors 2, 3, and 4 each accounted for 12% of the total variance and had rotated eigenvalues of 2.20, 2.19 and 2.15. Factor 2 exhibited loadings from .48 to .95, with communalities ranging from .44 and .76. Factor 3 displayed loadings from .45 to .91, with communalities between .44 and .64, and Factor 4 featured loadings from .38 to .69, with communalities from .39 to .58.

**Table 6:** Exploratory factor analysis of 18 PIQ-items with promax rotation.

	Factor loadings				Communality
	1	2	3	4	
Emotional distress					
IP1: I am mistreated more often than other people.	.64				.39
EEC6: I feel guilty and/or ashamed.	.52			-.34	.31
EEC7: I feel humiliated.	.52				.57
EEC4: I feel left alone.	.52				.42
IP3: I often get less than I deserve.	.51				.36
EEC3: I feel betrayed by humanity and cannot trust many people.	.51				.45
Fear of recurrence					
EEC2: I feel unsafe.		.95			.76
IE2: I feel that acts of injustice that I have had experienced affected me in a permanent way.		.58			.59
EEC1: I am scared that I will experience injustice again in my life.		.53			.55
IP5: It all seems so unfair.		.48			.44
Revenge and anger					
RF3: I do not want to forgive, I want revenge.			.91		.64
RF2: I want to punish the person who has hurt me.			.77		.58
EEC5: I feel a lot of anger, range or aggression.			.59		.55
RF4: I cannot forgive the people who did not help me.			.45		.44
Perceived disadvantage					
IP2: My life is a lot harder than the lives of people around me.				.69	.47
IE3: Some of the experiences that I have had were a lot worse than what humans generally experience.				.68	.44
RF1: Nothing will ever make up for what I have gone through.				.58	.58
IP4: I am suffering because of someone else’s negligence.				.38	.39
Variance explained	13%	12%	12%	12%	
Rotated eigenvalues	2.38	2.20	2.19	2.15	

An Oblimin rotation yielded similar results with slightly weaker correlations and no substantial changes in factor loadings. Exploratory hierarchical factor analysis with a general factor resulted in a less favorable model fit (RMSR = .10, RMSEA = .10, BIC = -311.32) and failed to provide a clearer structure.

Factor 1, *Emotional Distress*, consisted of items reflecting emotional suffering as a consequence of perceived injustice, including feelings of guilt, shame, humiliation, isolation, betrayal, and perceived mistreatment. Factor 2, *Fear of Recurrence*, comprised items related to anxiety and concerns about the temporal aspect of perceived injustice, including feelings of insecurity, fear of future injustice, perceptions of ongoing unfairness, and apprehensions about the lasting effects of past injustices. Factor 3, *Revenge and Anger*, included items indicative of a desire for revenge, feelings of anger, aggression, and an inability to forgive. Factor 4, *Perceived Disadvantage*, was characterized by items relating to perceiving one’s life as more challenging than others, believing that one’s experiences have been notably worse, and the belief that nothing can compensate for past suffering. The cross-loading of ‘EEC6’ on Factor 1 (.52) and factor 4 (-.34) highlights the interplay between internal emotional distress and external perceptions of disadvantage. This suggests that individuals experiencing guilt and shame (internal attributions of distress) may be less likely to focus on external factors, such as perceiving their situation as worse compared to others.

#### 3.6.1 Reliability

The overall questionnaire displayed excellent overall reliability (α = .90). Among the subscales, the Fear of Recurrence and Revenge and Anger scales exhibited good reliability (α = .81 and α = .80), while the Emotional Distress and Perceived Disadvantage scales displayed moderate reliability (α = .78 and α = .74). Therefore, hypothesis 2, which posited that both the overall test and the subscales of the Ukrainian version would demonstrate good reliability, was only partially confirmed.

#### 3.6.2 Criterion validity

The PIQ showed significant positive correlations with all mental health measures (**Table 7**). The strongest association was observed with PTSD symptoms (PCL-5, r = .71, *p* <.01), followed by depression (PHQ-9, r = .62, *p* <.01) and anxiety (GAD-7, r = .61, *p* <.01). The weakest correlation was found with war-related trauma (LEC-5, r = .35, *p* <.01). These results support hypothesis 3 and 4, which posited that perceived injustice relates to exposure to potentially traumatic events as well as various mental health symptoms.

**Table 7:** Correlations between perceived injustice and mental disorder symptoms.

Variable	1	2	3	4
1. PIQ				
2. LEC-5	.35**			
	[.21,.47]			
3. PHQ-9	.62**	.30**		
	[.52,.70]	[.16,.43]		
4. PCL-5	.71**	.38**	.76**	
	[.63,.78]	[.25,.50]	[.68,.81]	
5. GAD-7	.61**	.32**	.74**	.78**
	[.51,.70]	[.18,.45]	[.67,.80]	[.71,.83]

PIQ, Perceived Injustice Questionnaire; LEC-5,Life Events Checklist for DSM-5; PHQ-9, Patient Health Questionnaire-9; PCL-5, Posttraumatic Stress Disorder Checklist for DSM-5; GAD-7, Generalized Anxiety Disorder 7-item scale. Values in square brackets indicate the 95% confidence interval for each correlation. ** indicates *p* <.01.

Hierarchical multiple regression analyses indicated that PTLEs significantly predicted depression scores (*β* = .30, *t*(168) = 4.07, *p* <.01), accounting for 9% of the variance (*R^2^* = .09, *F*(1, 168) = 16.58, *p* <.01). With the addition of PIQ as a predictor, a significant improvement in model fit was observed (*R^2^* = .39, *F*(2, 167) = 53.56, p <.01), with PIQ uniquely explaining 33% of the variance (Partial *R^2^* = .33, Δ*R²* = .30). Furthermore, PTLE lost statistical significance in the presence of PIQ. The change in model fit was significant (ΔRSS = 50.86, *F*(1,167) = 82.50, p <.01), indicating that the inclusion of PIQ significantly enhanced the model’s ability to explain variance in depression scores (**Table 8**).

**Table 8:** Hierarchical multiple regression results with PHQ-9 as the criterion.

Predictor	*β*	sr^2^	Fit	Difference
LEC-5	0.30** [0.15, 0.45]	.09 [.02,.18]		
			*R^2^* = .090**	
			95% CI[.02,.18]	
LEC-5	0.10 [-0.03, 0.22]	.01 [-.01,.03]		
PIQ	0.59** [0.46, 0.71]	.30 [.19,.41]		
			*R^2^* = .391**	Δ*R^2^* = .301**
			95% CI[.27,.48]	95% CI[.19,.41]

LEC-5, Life Events Checklist for DSM-5; PIQ, Perceived Injustice Questionnaire; PHQ-9, Patient Health Questionnaire-9. The intercept is fixed at zero in all models. *β* represents the standardized regression weights. *sr^2^* represents the semi-partial correlation squared. Values in square brackets indicate the 95% confidence interval. * indicates *p* <.05. ** indicates *p* <.01.

For generalized anxiety disorder, hierarchical multiple regression analysis indicated that PTLEs also predicted anxiety scores, *β* = .32, *t*(168) = 4.40, *p* <.01, explaining 11% of the variance (*R²* = .11, *F*(1, 168) = 19.71, *p* <.01). The inclusion of PIQ significantly improved the model fit (*R²* = .39, *F*(2, 167) = 52.52, *p* <.01), with PIQ contributing uniquely to 31% of the variance (Partial *R²* = .31, *ΔR²* = .28). Similar to the depression model, LEC-5 lost significance post-PIQ inclusion (**Table 9**).

**Table 9:** Hierarchical multiple regression results with GAD-7 as the criterion.

Predictor	*β*	*sr^2^*	Fit	Difference
LEC-5	0.32** [0.18, 0.47]	.10 [.03,.20]		
			*R^2^* = .105**	
			95% CI[.03,.20]	
LEC-5	0.13 [-0.00, 0.25]	.01 [-.01,.04]		
PIQ	0.57** [0.44, 0.69]	.28 [.17,.39]		
			*R^2^* = .386**	Δ*R^2^* = .281**
			95% CI[.27,.48]	95% CI[.17,.39]

LEC-5, Life Events Checklist for DSM-5; PIQ, Perceived Injustice Questionnaire; GAD-7, Generalized Anxiety Disorder 7-item scale. The intercept is fixed at zero in all models. *β* represents the standardized regression weights. *sr^2^* represents the semi-partial correlation squared. Values in square brackets indicate the 95% confidence interval. ** indicates *p* <.01.

For PTSD, hierarchical multiple regression analysis demonstrated that PTLEs also significantly predicted PTSD scores (*β* = 0.38, *t*(168) = 5.38, *p* <.01), explaining 15% of the variance (*R²* = .15, *F*(1, 168) = 28.94, *p* <.01). The inclusion of PIQ substantially improved model fit (*R²* = .53, *F*(2, 167) = 93.38, *p* <.01), with PIQ contributing uniquely to 45% of the variance (Partial *R²* = .45, *ΔR²* = .38). Notably, LEC-5 remained statistically significant post-PIQ inclusion, distinguishing it from the depression and anxiety models (**Table 10**).

**Table 10:** Hierarchical multiple regression results with PCL-5 as the criterion.

Predictor	*β*	*sr^2^*	Fit	Difference
LEC-5	0.38** [0.24, 0.52]	.15 [.06,.24]		
			*R^2^* = .147**	
			95% CI[.06,.24]	
LEC-5	0.15** [0.04, 0.27]	.02 [-.01,.05]		
PIQ	0.66** [0.55, 0.77]	.38 [.27,.49]		
			*R^2^* = .528**	Δ*R^2^* = .381**
			95% CI[.42,.60]	95% CI[.27,.49]

LEC-5, Life Events Checklist for DSM-5; PIQ, Perceived Injustice Questionnaire; PCL-5, Posttraumatic Stress Disorder Checklist for DSM-5. The intercept is fixed at zero in all models. *β* represents the standardized regression weights. *sr^2^* represents the semi-partial correlation squared. Values in square brackets indicate the 95% confidence interval. * indicates *p* <.05. ** indicates *p* <.01.

## 4 Discussion

### 4.1 Construct validity

The analysis revealed a four-factor model in the Ukrainian version, with factors capturing Emotional Distress, Fear of Recurrence, Revenge and Anger, and Perceived Disadvantage. Hereby, the Emotional Distress (ED) factor, closely resembled the original Emotional and Cognitive Consequences (EEC) factor. While most items from the EEC factor remained within Emotional Distress, three items found new associations. Additionally, two Injustice Perception (IP) items were added to the ED factor, highlighting its link to perceived social injustice. Unlike the original questionnaire, the Ukrainian version no longer presented Injustice Perception and Injustice Experience as distinct factors. Instead, items from these factors contributed to the formation of Fear of Recurrence and Perceived Disadvantage constructs. The Revenge and Anger factor in the Ukrainian version closely resembled the original Revenge and Forgiveness (RF) factor, incorporating three RF items and one additional item related to anger from the EEC factor. The differences in factor structure between the Ukrainian and English versions may suggest various biases in cross-cultural assessment.

#### 4.1.1 Method bias

The observed differences in factor structures between the Ukrainian version and the original version of the questionnaire could be partly attributed to differences in the samples and analysis methods between the studies.The sample size of 89 participants in the original study might have restricted the stability of the factor structure. In addition, the presence of cross- and double-loadings could have led to uncertainty in item-factor relationships. Additionally, the choice of Principal Component Analysis (PCAmight have affected the validity, as Exploratory Factor Analysis (EFA) could have provided a more robust framework.

Sampling effects, including demographic variations and conflict- and flight-specific influences, could also contribute to the differences. The original study consisted of Northern Iraqi students, while the current study included Ukrainians with varying ages and educational backgrounds. To examine this assumption, one approach could be to compare the Ukrainian sample with more diverse Northern Iraq samples, including Iraqi refugees in camps, expatriate Iraqis, and those who remained in their hometowns throughout the conflict. Another approach could involve assessing group differences in flight experience and its impact on perceived injustice.

Response bias might also have influenced the study. Different cultural backgrounds could have lead to distinct response styles (77). To address this bias, future research could employ randomized response techniques and consider scaling direction by comparing responses in both ascending and descending order.

#### 4.1.2 Item bias

Despite evidence-based translation, the lack of structural equivalence may be attributed to specific items disrupting the factor structure. Comparing item difficulties between the Ukrainian and original versions could be an initial step in exploring this issue. Analyzing item difficulties reveals variations in response patterns across diverse cultural backgrounds (78). Additionally, employing Differential Item Functioning (DIF) within the framework of multiple-group CFA could further investigate item bias and its impact on the factor structure (79).

#### 4.1.3 Construct bias

The factorial structure of the questionnaire might also have been influenced by the diverse cultural backgrounds of Ukrainians and Northern Iraqis. Ukrainians, with its history of political tensions and struggles for independence, may have prioritized themes like sovereignty and autonomy in justice perceptions, particularly amplified by recent conflicts. In contrast, Northern Iraqi culture, characterized by ethno-religious diversity and conflict legacies, might have offered a broader perspective on justice influenced by historical grievances and religious beliefs.

Deviations from the original version pose both challenges and opportunities. On the one hand, it raises concerns about measurement equivalence and the appropriateness of cross-cultural comparisons using the same questionnaire. Simultaneously, the lack of equivalence could also signal systematic cross-cultural differences, offering valuable insights into the interplay between culture and psychological assessments. This diversity in factor structures might be viewed as a valuable source of information, deepening the understanding of justice perceptions and providing insights into the multifaceted nature of justice across various cultural contexts. A thorough research strategy, including item analysis and evaluation of measurement equivalence, is necessary to determine if cross-cultural comparisons are feasible or if culturally sensitive versions are preferable. Comparing various language versions across Schwartz’s cultural regions could be a potential framework (78, 80).

While direct cross-cultural comparisons using the PIQ are currently unfeasible, there are indications of functional equity between the two existing versions. Each language version may independently hold merit while sharing similar correlations with related constructs. Establishing unique cutoff scores for each version is crucial, potentially referencing the mean score (e.g., 2.5) or aligning these thresholds with the PIQ’s correlation to symptom severity in conditions such as depression, PTSD, and anxiety. This nuanced approach would acknowledge the potential strengths of each version while recognizing the necessity for tailored assessment standards.

### 4.2 Reliability

The Ukrainian version of the test showed excellent overall reliability, though subscale reliability varied. This underscores the need to scrutinize individual subscale items to boost reliability and questionnaire precision. Therefore, while the overall PIQ can be used to assess clinically significant levels of perceived injustice, practitioners should consider the sub-scales primarily only as indicators and guides to perceived injustice quality, refraining from drawing direct treatment decisions based on them.

Additionally, given the moderate reliability of some subscales and the substantial overlap between the factors, it may be worth considering the development of an unidimensional, more concise version of the questionnaire. A shorter form could offer several advantages, like reducing respondent burden, potentially increasing participation rates, and improving the questionnaire’s practicality in both clinical and research settings.

### 4.3 Criterium validity

Our findings indicated a weak correlation between potentially traumatic life events and the PIQ, a moderate correlation between depression and generalized anxiety disorder symptoms, and the PIQ, and a strong correlation between PTSD symptoms and the PIQ. Possible explanations for these trends will be discussed in the following.

#### 4.3.1 Perceived injustice and PTLEs

The weak correlation between perceived injustice and potentially traumatic life events might stem from varying individual interpretations of trauma’s impact (81, 82). Traumatic experiences are subjective, and how they are perceived can differ greatly among individuals (83). Additionally, while various models explain trauma processing, little is understood about how perceived injustice arises in response to trauma, presenting a research opportunity (e.g., 84–86).

#### 4.3.2 Perceived injustice and depression

The moderate correlation between the PIQ and depression might be ascribed to common elements, such as victimization and unfairness, which hold a central position in the development of depressive symptoms (87). Furthermore, as outlined by Sullivan et al. (88), perceived injustice can lead to anger and prolonged emotional responses (89), as well as potentially challenging therapist-client alliances (90). Attributions of blame and retribution motives from perceived injustice may increase social conflict and lead to invalidating responses in the depressed individual’s social environment (91). These experiences can lead to social withdrawal and contribute to the persistence of depressive symptoms (92).

#### 4.3.3 Perceived injustice and generalized anxiety disorder

The moderate correlation between perceived injustice and generalized anxiety disorder symptoms becomes clearer in light of recent research on trait justice sensitivity (JS). JS is a stable individual disposition marked by heightened sensitivity to perceiving and strongly reacting to situations involving injustice (93, 94). This trait is closely tied to various emotional responses, including feelings of helplessness, sadness, social withdrawal, and an increased fear of future victimization (95). Individuals with high trait JS often struggle to downplay or rationalize unfair treatment, resulting in heightened emotional intensity and anticipatory fear concerning justice-related situations (96). These traits bear a striking resemblance to the experiences of individuals with anxiety disorders, who frequently engage in repetitive negative appraisals of past social encounters and often exhibit negative affect and cognition (97). The correlation between perceived injustice and generalized anxiety disorder symptoms can be partially understood by viewing perceived injustice as a transient state akin to JS, triggered by traumatic experiences. Such experiences may sensitize individuals to perceived injustice, evoking heightened emotional responses, which can subsequently manifest as symptoms of anxiety disorders.

#### 4.3.4 Perceived injustice and PTSD

The substantial correlation between the PIQ and PTSD symptoms underscores the alignment with conceptual models proposing a causative link between maladaptive cognitive processes and the development of post-traumatic stress symptoms. This includes the amplifying effects of ruminative thinking on the emotional impact of traumatic war and flight experiences (98). Furthermore, cognitive factors related to the perception of irreparability and loss emerge as potential pathways through which perceived injustice perpetuates ongoing PTSD symptoms, as indicated by Sullivan et al. (22). Moreover, although the field is still in the early stages of exploring this relationship, emerging research highlights the significance of trait anger and its expression as potential mechanisms linking perceived injustice with the emergence of post-traumatic stress symptoms (99). This aligns with established literature emphasizing the frequent co-occurrence of anger expression as a notable feature of PTSD (100).

Systematic research is essential for developing a robust conceptual model of perceived injustice and its relationship with PTSD, depression, and generalized anxiety disorder. A promising starting point would involve a thorough investigation of the specific facets of the construct. Based on the outlined research, for instance, the factor Revenge and Anger might exhibit the strongest correlation with PTSD, while the factor Fear of Recurrence could be closely linked to anxiety disorders.

### 4.4 Incremental validity

Potentially traumatic life events explained 9% and 11% of the variance in depression and generalized anxiety disorder symptoms, suggesting a limited impact. However, incorporating perceived injustice significantly improved both models, explaining 39% of the variance in both conditions. Perceived injustice alone accounted for 33% of depression symptoms and 31% of generalized anxiety disorder symptoms, surpassing the predictive value of traumatic life events and highlighting its strong influence.

In PTSD, potentially traumatic life events explained 15% of the variance, a moderate effect size consistent with PTSD’s symptomatology, closely linked to the occurrence of flashbacks from traumatic experiences (101). However, integrating perceived injustice significantly improved the model, with both factors jointly explaining 53% of the variance. Perceived injustice independently accounted for 45%, highlighting its strong predictive power. Notably, traumatic life events retained statistical significance even after considering perceived injustice, emphasizing the importance of both factors in predicting PTSD.

### 4.5 Significance for science and clinical practice

The study’s findings underscore the PIQ’s potential for identifying clinically significant levels of perceived injustice, which are closely associated with symptoms of depression, generalized anxiety disorder, and PTSD in individuals affected by war-related trauma and displacement. Integrating the PIQ into clinical practice offers a nuanced understanding of perceived injustice, facilitating targeted treatment and support tailored to individual experiences. Additionally, these results highlight the need for psychotherapeutic interventions addressing perceived injustice, paving the way for evidence-based approaches to support trauma-affected individuals. Future research should focus on developing a comprehensive cognitive model of perceived injustice, exploring its connections with other psychological constructs to enhance theoretical understanding and inform clinical interventions.

### 4.6 Limitations

The study is subject to some limitations. First, the sample composition, predominantly comprising females, individuals with higher education levels, and those who fled abroad, may restrict the generalizability of findings to the broader Ukrainian population. Future studies should aim for a more diverse sample, particularly from within Ukraine. Furthermore, recruiting participants primarily through activists might have introduced selection bias, as these individuals may exhibit a heightened focus on justice compared to the general population. Diversifying recruitment channels can help mitigate this bias. Moreover, offering only a Ukrainian language version of the PIQ might have introduced language bias, especially considering the linguistic diversity in Ukraine. Providing a Russian-language version, particularly in Eastern Ukraine, could address this issue. The inadvertent omission of one item during the translation process might have impacted the factor structure. Re-analysis, including this item, is warranted, considering its potential significance. Additionally, the study did not examine the temporal stability of perceived injustice, leaving its evolution over time unexplored. Investigating this, possibly in conjunction with trait justice sensitivity, could provide valuable insights. Lastly, exploring changes in perceived injustice before and after conflicts could elucidate its cultural dynamics and societal role, offering valuable insights into its evolution and significance.

## 5 Conclusion

The study contributes to understanding Ukrainian perceptions of injustice, providing insights into the unique factors shaping their experiences. Additionally, it establishes the Perceived Injustice Questionnaire as a reliable tool for assessing perceived injustice, which is closely linked to trauma-related disorders. The demonstrated criterion validity calls for its integration into therapeutic interventions to address the impact of perceived injustice on mental health. Through the PIQ, individuals who have developed a strong sense of injustice due to war and displacement can be identified, allowing for a focus on this aspect in their psychotherapy. In the long term, therapy modules addressing perceived injustice should be developed and implemented. Future research avenues also should include exploring the relationship between perceived injustice and other psychological constructs, developing a comprehensive cognitive model, and further refining its cross-cultural applicability.

## Data Availability

The raw data supporting the conclusions of this article will be made available by the authors, without undue reservation.

## References

[1] United Nations. ‘Dire year’ for children caught in conflict, as hospital and school attacks double: UN News (2023). Available online at: https://news.un.org/en/story/2023/06/1138137 (Accessed September 29, 2023).

[2] Human Rights Watch. Ukraine: Russians pillage Kherson cultural institutions Human Rights Watch (2022). Available online at: https://www.hrw.org/news/2022/12/20/Ukraine-Russians-pillage-kherson-cultural-institutions (Accessed September 29, 2023).

[3] Steel Z Chey T Silove D Marnane C Bryant RA van Ommeren M. Association of torture and other potentially traumatic events with mental health outcomes among populations exposed to mass conflict and displacement: a systematic review and meta-analysis. Jama. (2009) 302:537–49. doi: 10.1001/jama.2009.1132 19654388

[4] Murthy RS Lakshminarayana R. Mental health consequences of war: a brief review of research findings. World Psychiatry. (2006) 5:25–30. 16757987 PMC1472271

[5] Blackmore R Boyle JA Fazel M Ranasinha S Gray KM Fitzgerald G. The prevalence of mental illness in refugees and asylum seekers: A systematic review and meta-analysis. PloS Med. (2020) 17:e1003337. doi: 10.1371/journal.pmed.1003337 32956381 PMC7505461

[6] World Health Organization. Mental disorders affect one in four people (2001). Available online at: https://www.who.int/news/item/28-09-2001-the-world-health-report-2001-mental-disorders-affect-one-in-four-people (Accessed September 29, 2023).

[7] Bromet EJ Gluzman SF Paniotto VI Webb CP Tintle NL Zakhozha V. Epidemiology of psychiatric and alcohol disorders in Ukraine: findings from the Ukraine World Mental Health survey. Soc Psychiatry Psychiatr Epidemiol. (2005) 40:681–90. doi: 10.1007/s00127-005-0927-9 16160752

[8] World Bank Group. Mental health in transition: Assessment and guidance for strengthening integration of mental health into primary health care and community-based service platforms in Ukraine. Washington, D.C.: World Bank Group (2017) Available online at: http://documents.worldbank.org/curated/en/310711509516280173/Mental-health-in-transition-assessment-and-guidance-for-strengthening-integration-of-mental-health-into-primary-health-care-and-community-based-service-platforms-in-Ukraine (Accessed September 29, 2023).

[9] Korostiy V. Mental health of internally displaced persons in Ukraine. Eur Neuropsychopharmacol. (2016) 26:S633. doi: 10.1016/S0924-977X(16)31727-8

[10] Ramachandran A Makhashvili N Javakhishvili J Karachevskyy A Kharchenko N Shpiker M. Alcohol use among conflict-affected persons in Ukraine: risk factors, coping and access to mental health services. Eur J Public Health. (2019) 29:1141–6. doi: 10.1093/eurpub/ckz117 31230084

[11] Roberts B Makhashvili N Javakhishvili J Karachevskyy A Kharchenko N Shpiker M. Mental health care utilisation among internally displaced persons in Ukraine: results from a nation-wide survey. Epidemiol Psychiatr Sci. (2019) 28:100–11. doi: 10.1017/S2045796017000385 PMC6998949 28747237

[12] Ben-Ezra M Goodwin R Leshem E Hamama-Raz Y. PTSD symptoms among civilians being displaced inside and outside the Ukraine during the 2022 Russian invasion. Psychiatry Res. (2023) 320:115011. doi: 10.1016/j.psychres.2022.115011 36566594

[13] Karatzias T Shevlin M Ben-Ezra M McElroy E Redican E Vang ML. War exposure, posttraumatic stress disorder, and complex posttraumatic stress disorder among parents living in Ukraine during the Russian war. Acta Psych. Scandinavica. (2023) 147:276–85. doi: 10.1111/acps.13529 36625445

[14] Zasiekina L Zasiekin S Kuperman V. Post-traumatic stress disorder and moral injury among Ukrainian civilians during the ongoing war. J Community Health. (2023) 48(5):784–92. doi: 10.1007/s10900-023-01225-5 37119352 PMC10148618

[15] Chudzicka-Czupała A Hapon N Chiang SK Żywiołek-Szeja M Karamushka L Lee CT. Depression, anxiety and post-traumatic stress during the 2022 Russo-Ukrainian war, a comparison between populations in Poland, Ukraine, and Taiwan. Sci Rep. (2023) 13:3602. doi: 10.1038/s41598-023-28729-3 36869035 PMC9982762

[16] Kurapov A Danyliuk I Loboda A Kalaitzaki A Kowatsch T Klimash T. Six months into the war: a first-wave study of stress, anxiety, and depression among in Ukraine. Front Psychiatry. (2023) 14:1190465. doi: 10.3389/fpsyt.2023.1190465 37234208 PMC10206008

[17] Xu W Pavlova I Chen X Petrytsa P Graf-Vlachy L Zhang SX. Mental health symptoms and coping strategies among Ukrainians during the Russia-Ukraine war in March 2022. Int J Soc Psychiatry. (2023) 69:957–66. doi: 10.1177/00207640221143919 36598090

[18] Johnson RJ Antonaccio O Botchkovar E Hobfoll SE. War trauma and PTSD in Ukraine’s civilian population: comparing urban-dwelling to internally displaced persons. Soc Psychiatry Psychiatr Epidemiol. (2022) 57(9):1807–16. doi: 10.1007/s00127-021-02176-9 34596712

[19] Carriere JS Donayre Pimentel S Yakobov E Edwards RR. A systematic review of the association between perceived injustice and pain-related outcomes in individuals with musculoskeletal pain. Pain Med. (2020) 21:1449–63. doi: 10.1093/pm/pnaa088 32377686

[20] Ezenwa MO Molokie RE Wilkie DJ Suarez ML Yao Y. Perceived injustice predicts stress and pain in adults with sickle cell disease. Pain Manage Nursing. (2015) 16:294–306. doi: 10.1016/j.pmn.2014.08.004 PMC4417102 25439119

[21] Lynch J Fox S D’Alton P Gaynor K. A systematic review and meta-analysis of the association between perceived injustice and depression. J Pain. (2021) 22:643–54. doi: 10.1016/j.jpain.2020.12.009 33465504

[22] Sullivan MJ Thibault P Simmonds MJ Milioto M Cantin A-P Velly AM. Pain, perceived injustice and the persistence of post-traumatic stress symptoms during the course of rehabilitation for whiplash injuries. Pain. (2009) 145:325–31. doi: 10.1016/j.pain.2009.06.031 19643543

[23] Francis L Barling J. Organizational injustice and psychological strain. Can J Behav Science/Revue Can Des Sci du comportement. (2005) 37:250–61. doi: 10.1037/h0087260

[24] Rousseau V Salek S Aubé C Morin EM. Distributive justice, procedural justice, and psychological distress: The moderating effect of coworker support and work autonomy. J Occup Health Psychol. (2009) 14:305–17. doi: 10.1037/a0015747 19586224

[25] Tatar JR II Kaasa SO Cauffman E. Perceptions of procedural justice among female offenders: Time does not heal all wounds. Psychol Public Policy Law. (2012) 18:268–96. doi: 10.1037/a0025118

[26] Grote NK Clark MS Moore A. Perceptions of injustice in family work: the role of psychological distress. J Family Psychol. (2004) 18:480–92. doi: 10.1037/0893-3200.18.3.480 PMC3025776 15382973

[27] Pham PN Weinstein HM Longman T. Trauma and PTSD symptoms in Rwanda: implications for attitudes toward justice and reconciliation. Jama. (2004) 292:602–12. doi: 10.1001/jama.292.5.602 15292086

[28] Tay AK Rees SJ Tam N Savio E Costa ZMD Silove D. The role of trauma-related injustice in pathways to posttraumatic stress symptoms among conjugal couples: A multilevel, dyadic analysis in postconflict Timor-Leste. SAGE Open. (2017) 7:1–10. doi: 10.1177/2158244017723688

[29] Başoglu M Livanou M Crnobarić C Francisković T Suljić E Durić D. Psychiatric and cognitive effects of war in former yugoslavia: association of lack of redress for trauma and posttraumatic stress reactions. Jama. (2005) 294:580–90. doi: 10.1001/jama.294.5.580 16077052

[30] Neumann JC Berger T Kizilhan JI. Development of a questionnaire to measure the perceived injustice of people who have experienced violence in war and conflict Areas: Perceived Injustice Questionnaire (PIQ). Int J Environ Res Public Health. (2021) 18(23):12357. doi: 10.3390/ijerph182312357 34886079 PMC8657181

[31] Tsang S Royse C Terkawi A. Guidelines for developing, translating, and validating a questionnaire in perioperative and pain medicine. Saudi J Anesth. (2017) 11:80–9. doi: 10.4103/sja.SJA_203_17 PMC5463570 28616007

[32] Beavers AS Lounsbury JW Richards JK Huck SW Skolits GJ Esquivel SL. Practical considerations for using exploratory factor analysis in educational research. Pract Assess Res Eval. (2013) 18:6. doi: 10.7275/

[33] Suhr D. Exploratory or Confirmatory Factor Analysis? Proceedings of the 31st Annual SAS? Users Group International Conference. Cary, NC: SAS Institute Inc., Paper Number: 200-31. (2006). Available online at: https://citeseerx.ist.psu.edu/document?repid=rep1&type=pdf&doi=915520bdfde1423b0b73f7ed560c68e81678cda6

[34] Faul F Erdfelder E Lang A-G Buchner A. G* Power 3: A flexible statistical power analysis program for the social, behavioral, and biomedical sciences. Behav Res Methods. (2007) 39:175–91. doi: 10.3758/BF03193146 17695343

[35] Maeda H. Response option configuration of online administered Likert scales. Int J Soc Res Methodol. (2015) 18:15–26. doi: 10.1080/13645579.2014.885159

[36] Liu M Keusch F. Effects of scale direction on response style of ordinal rating scales. J Off Stat. (2017) 33:137–54. doi: 10.1515/jos-2017-0008

[37] Weathers F Blake D Schnurr P Kaloupek D Marx B Keane T. The life events checklist for DSM-5 (LEC-5). Instrument available from the National Center for PTSD (2013). Available online at: https://www.ptsd.va.gov.

[38] Pugach CP Nomamiukor FO Gay NG Wisco BE. Temporal stability of self-reported trauma exposure on the life events checklist for DSM-5. J Trauma Stress. (2021) 34:248–56. doi: 10.1002/jts.22611 33089510

[39] Contractor AA Weiss NH Natesan P Elhai JD. Clusters of trauma types as measured by the life events checklist for DSM-5. Int J Stress Manage. (2020) 27:380–93. doi: 10.1037/str0000179 PMC8932936 35311212

[40] Gray MJ Litz BT Hsu JL Lombardo TW. Psychometric properties of the life events checklist. Assessment. (2004) 11:330–41. doi: 10.1177/1073191104269954 15486169

[41] The International Trauma Consortium. Mental Health Resources for Clinicians and Researchers . Available online at: https://www.traumameasuresglobal.com/_files/ugd/be25b4_7abaa23347d24234be7f6f1b0f6c3bf4.docx?dn=2.%20Trauma%20Exposure%20LEC5_Ukrainian.docx (Accessed September 29, 2023).

[42] Kroenke K Spitzer RL Williams JB. The PHQ-9: validity of a brief depression severity measure. J Gen Internal Med. (2001) 16:606–13. doi: 10.1046/j.1525-1497.2001.016009606.x PMC1495268 11556941

[43] Kroenke K Spitzer RL Williams JB Löwe B. The Patient Health Questionnaire Somatic, Anxiety, and Depressive Symptom Scales: a systematic review. Gen Hosp Psychiatry. (2010) 32:345–59. doi: 10.1016/j.genhosppsych.2010.03.006 20633738

[44] Martin A Rief W Klaiberg A Braehler E. Validity of the Brief Patient Health Questionnaire Mood Scale (PHQ-9) in the general population. Gen Hosp Psychiatry. (2006) 28:71–7. doi: 10.1016/j.genhosppsych.2005.07.003 16377369

[45] Hyland P Vallières F Shevlin M Karatzias T Ben-Ezra M McElroy E. Psychological consequences of war in Ukraine: Assessing changes in mental health among Ukrainian parents. psychol Med. (2023) 53(15):7466–8. doi: 10.1017/S0033291723000818 37016786 PMC10719667

[46] Blevins CA Weathers FW Davis MT Witte TK Domino JL. The posttraumatic stress disorder checklist for DSM-5 (PCL-5): development and initial psychometric evaluation. J Traumatic Stress. (2015) 28:489–98. doi: 10.1002/jts.2015.28.issue-6 26606250

[47] Bovin MJ Marx BP Weathers FW Gallagher MW Rodriguez P Schnurr PP. Psychometric properties of the PTSD Checklist for Diagnostic and Statistical Manual of Mental Disorders-Fifth Edition (PCL-5) in veterans. Psychol Assess. (2016) 28:1379–91. doi: 10.1037/pas0000254 26653052

[48] Shevlin M Hyland P Vallières F Bisson J Makhashvili N Javakhishvili J. A comparison of DSM-5 and ICD-11 PTSD prevalence, comorbidity and disability: an analysis of the Ukrainian Internally Displaced Person’s Mental Health Survey. Acta Psychiatr Scand. (2018) 137:138–47. doi: 10.1111/acps.2018.137.issue-2 29210054

[49] Spitzer RL Kroenke K Williams JB Löwe B. A brief measure for assessing generalized anxiety disorder: the GAD-7. Arch Internal Med. (2006) 166:1092–7. doi: 10.1001/archinte.166.10.1092 16717171

[50] Löwe B Decker O Müller S Brähler E Schellberg D Herzog W. Validation and standardization of the Generalized Anxiety Disorder Screener (GAD-7) in the general population. Med Care. (2008) 46:266–74. doi: 10.1097/MLR.0b013e318160d093 18388841

[51] LimeSurvey GmbH. LimeSurvey: An Open Source survey tool Hamburg, Germany. Hamburg: LimeSurvey GmbH. Available at: http://www.limesurvey.org (Accessed September 29, 2024).

[52] Berufsethische Richtlinien des Berufsverbandes Deutscher Psychologinnen und Psychologen eV und der Deutschen Gesellschaft für Psychologie eV Berlin. Berlin: Föderation Deutscher Psychologenvereinigungen GbR. (2022).

[53] R Core Team. R: A language and environment for statistical computing. Vienna, Austria: R Foundation for Statistical Computing (2022).

[54] Finney SJ DiStefano C. Non-normal and categorical data in structural equation modeling. In: Hancock GR Mueller RO (Eds.). Structural equation modeling: A second course, 2nd ed. Charlotte: IAP Information Age Publishing. (2006). p. 269–314.

[55] Hu L-t Bentler PM. Fit indices in covariance structure modeling: Sensitivity to underparameterized model misspecification. psychol Methods. (1998) 3:424–53. doi: 10.1037/1082-989X.3.4.424

[56] Hu L-t Bentler PM. Cutoff criteria for fit indexes in covariance structure analysis: Conventional criteria versus new alternatives. Struct equation modeling: Multidiscip J. (1999) 6:1–55. doi: 10.1080/10705519909540118

[57] Cho G Hwang H Sarstedt M Ringle CM. Cutoff criteria for overall model fit indexes in generalized structured component analysis. J Market Anal. (2020) 8:189–202. doi: 10.1057/s41270-020-00089-1

[58] Iacobucci D. Structural equations modeling: Fit indices, sample size, and advanced topics. J consumer Psychol. (2010) 20:90–8. doi: 10.1016/j.jcps.2009.09.003

[59] DiStefano C Liu J Jiang N Shi D. Examination of the weighted root mean square residual: Evidence for trustworthiness? Struct Equation Model: A Multidiscip J. (2018) 25:453–66. doi: 10.1080/10705511.2017.1390394

[60] Velicer WF Eaton CA Fava JL. Construct explication through factor or component analysis: A review and evaluation of alternative procedures for determining the number of factors or components. In: Goffin RD Helmes E (Eds.), Problems and Solutions in Human Assessment. Boston: Springer. (2000). p. 41–71. doi: 10.1007/978-1-4615-4397-8_3

[61] Horn JL. A rationale and test for the number of factors in factor analysis. Psychometrika. (1965) 30:179–85. doi: 10.1007/BF02289447 14306381

[62] Velicer WF. Determining the number of components from the matrix of partial correlations. Psychometrika. (1976) 41:321–7. doi: 10.1007/BF02293557

[63] Klopp E. Using the BIC and sBIC to determine the number of factors in maximum-likelihood exploratory factor analysis. 14th Conference of the Expert Group on Methods and Evaluation of the German Psychological Society. Kiel (2019) doi: 10.17605/OSF.IO/Q7WHT.

[64] Tabachnick B Fidell L. Using multivariate statistics. 7th Еd. Boston: Allyn & Bacon/Pearson (2019).

[65] Hattori M Zhang G Preacher KJ. Multiple local solutions and geomin rotation. Multivariate Behav Res. (2017) 52:720–31. doi: 10.1080/00273171.2017.1361312 28952786

[66] Watkins M. A Step-by-Step Guide to Exploratory Factor Analysis with R and RStudio. New York/Abington: Routledge (2020).

[67] Brown TA. Confirmatory factor analysis for applied research. 2nd ed. New York, NY: Guilford Publications (2015).

[68] Finch WH. Using fit statistic differences to determine the optimal number of factors to retain in an exploratory factor analysis. Educ psychol Measure. (2020) 80:217–41. doi: 10.1177/0013164419865769 PMC7047263 32158020

[69] Comrey AL Lee HB. A first course in factor analysis. 2nd ed. New York: Psychology Press (1992).

[70] Norman GR Streiner DL. Biostatistics: the bare essentials. Hamilton: PMPH USA (BC Decker) (2008).

[71] Thurstone LL. Multiple factor analysis. psychol review. (1931) 38:406–27. doi: 10.1037/h0069792

[72] Sharif-Nia H She L Osborne J Gorgulu O Fomani FK Goudarzian AH. Statistical concerns, invalid construct validity, and future recommendations. Nurs Pract Today. (2024) 11:16–21. doi: 10.18502/npt.v11i1.14938

[73] DeVellis RF. Scale development: Theory and applications. 4th ed. Los Angeles: Sage (2017).

[74] Kline R. Efirmatory factor analysis. In: Petscher Y Schatschneider C Compton DL (Eds.). Applied quantitative analysis in the social sciences. New York, NY: Routledge (2013), p. 171–207.

[75] Benesty J Chen J Huang Y Cohen I. Pearson correlation coefficient. In: Noise reduction in speech processing. Springer, Heidelberg (2009). p. 37–40.

[76] Dancey CP Reidy J. Statistics without maths for psychology. Hoboken: Pearson Education (2007).

[77] Tellis GJ Chandrasekaran D. Does culture matter? Assessing response biases in cross-national survey research. Int J Res Market Forthcoming Marshall School Business Work Paper No MKT. (2010) 19-10. Available online at: https://www.researchgate.net/profile/Gerard-Tellis/publication/228135470_Does_Culture_Matter_Assessing_Response_Biases_in_Cross-National_Survey_Research/links/0c960521cd7c1f0001000000/Does-Culture-Matter-Assessing-Response-Biases-in-Cross-National-Survey-Research.pdf.

[78] De Beuckelaer A Lievens F Swinnen G. Measurement equivalence in the conduct of a global organizational survey across countries in six cultural regions. J Occup Organ Psychol. (2007) 80:575–600. doi: 10.1348/096317907X173421

[79] Kim G Chiriboga DA Jang Y. Cultural equivalence in depressive symptoms in older White, Black, and Mexican-American adults. J Am Geria Soc. (2009) 57:790–6. doi: 10.1111/j.1532-5415.2009.02188.x PMC3038683 19484834

[80] Schwartz SH. A theory of cultural values and some implications for work. Appl psychol: Int Rev. (1999) 48:23–47. doi: 10.1111/j.1464-0597.1999.tb00047.x

[81] Bower GH Sivers H. Cognitive impact of traumatic events. Dev Psychopathol. (1998) 10:625–53. doi: 10.1017/S0954579498001795 9886219

[82] Juczyński Z Ogińska-Bulik N. Cognitive processing of trauma as a predictor of the negative and positive consequences of experienced traumatic events. Adv Psychiatry Neurology/Postępy Psych i Neurol. (2018) 27:318–33. doi: 10.5114/ppn.2018.80884

[83] Norris FH. Epidemiology of trauma: frequency and impact of different potentially traumatic events on different demographic groups. J consult Clin Psychol. (1992) 60:409–18. doi: 10.1037/0022-006X.60.3.409 1619095

[84] Roussis P Wells A. Post-traumatic stress symptoms: Tests of relationships with thought control strategies and beliefs as predicted by the metacognitive model. Pers Individ Differ. (2006) 40:111–22. doi: 10.1016/j.paid.2005.06.019

[85] Liberzon I Abelson JL. Context processing and the neurobiology of post-traumatic stress disorder. Neuron. (2016) 92:14–30. doi: 10.1016/j.neuron.2016.09.039 27710783 PMC5113735

[86] Buckley TC Blanchard EB Neill WT. Information processing and ptsd: A review of the empirical literature. Clin Psychol Review. (2000) 20:1041–65. doi: 10.1016/S0272-7358(99)00030-6 11098399

[87] Scott W Sullivan MJL. Perceived injustice moderates the relationship between pain and depressive symptoms among individuals with persistent musculoskeletal pain. Pain Res Manage. (2012) 17:335–40. doi: 10.1155/2012/501260 PMC3465094 23061084

[88] Sullivan MJ Adams H Yamada K Kubota Y Ellis T Thibault P. The relation between perceived injustice and symptom severity in individuals with major depression: A cross-lagged panel study. J Affect Disord. (2020) 274:289–97. doi: 10.1016/j.jad.2020.05.129 32469818

[89] Darley JM Pittman TS. The psychology of compensatory and retributive justice. Pers Soc Psychol Review. (2003) 7:324–36. doi: 10.1207/S15327957PSPR0704_05 14650389

[90] Scott W Milioto M Trost Z Sullivan MJ. The relationship between perceived injustice and the working alliance: a cross-sectional study of patients with persistent pain attending multidisciplinary rehabilitation. Disabil rehabilitation. (2016) 38:2365–73. doi: 10.3109/09638288.2015.1129444 26805034

[91] Dickerson SS Gruenewald TL Kemeny ME. When the social self is threatened: Shame, physiology, and health. J Personal. (2004) 72:1191–216. doi: 10.1111/j.1467-6494.2004.00295.x 15509281

[92] Kool MB Geenen R. Loneliness in patients with rheumatic diseases: the significance of invalidation and lack of social support. J Psychol. (2012) 146:229–41. doi: 10.1080/00223980.2011.606434 22303622

[93] Bondü R. Is bad intent negligible? Linking victim justice sensitivity, hostile attribution bias, and aggression. Aggressive Behavior. (2018) 44:442–50. doi: 10.1002/ab.21764 29723423

[94] Baumert A Gollwitzer M Staubach M Schmitt M. Justice sensitivity and the processing of justice–related information. Eur J Personal. (2011) 25:386–97. doi: 10.1002/per.800

[95] Bondü R Holl AK Trommler D Schmitt MJ. Responses toward injustice shaped by justice sensitivity – evidence from Germany. Front Psychol. (2022) 13:858291. doi: 10.3389/fpsyg.2022.858291 36033064 PMC9399749

[96] Bondü R Inerle S. Afraid of injustice? Justice sensitivity is linked to general anxiety and social phobia symptoms. J Affect Disord. (2020) 272:198–206. doi: 10.1016/j.jad.2020.03.167 32553359

[97] Gkika S Wittkowski A Wells A. Social cognition and metacognition in social anxiety: A systematic review. Clin Psychol Psychother. (2018) 25:10–30. doi: 10.1002/cpp.v25.1 28836318

[98] Pavilanis A Truchon M Achille M Coté P Sullivan MJ. Perceived injustice as a determinant of the severity of post-traumatic stress symptoms following occupational injury. J Occup Rehab. (2023) 33:134–44. doi: 10.1007/s10926-022-10056-5 PMC10025196 35852696

[99] Trost Z Scott W Buelow MT Nowlin L Turan B Boals A. The association between injustice perception and psychological outcomes in an inpatient spinal cord injury sample: the mediating effects of anger. Spinal Cord. (2017) 55:898–905. doi: 10.1038/sc.2017.39 28555664

[100] Olatunji BO Ciesielski BG Tolin DF. Fear and loathing: A meta-analytic review of the specificity of anger in PTSD. Behav Ther. (2010) 41:93–105. doi: 10.1016/j.beth.2009.01.004 20171331

[101] Brewin CR. Re-experiencing traumatic events in PTSD: new avenues in research on intrusive memories and flashbacks. Eur J Psychotraumatol. (2015) 6:27180. doi: 10.3402/ejpt.v6.27180 25994019 PMC4439411

