# Supplementary material for: The Ukrainian version of the Perceived Injustice Questionnaire: A psychometric evaluation
Source: Front Psychiatry. 2024 Dec 2;15:1446724. doi: 10.3389/fpsyt.2024.1446724 (PMC11648219; doi:10.3389/fpsyt.2024.1446724)
Supplement: Supplementary file 1 [file Table1.docx]

**Appendix A**

**Perceived Injustice Questionnaire (PIQ)**

| **Please choose for each statement to what extent you agree with it.** | **Strongly agree** | **Agree** | **Neither agree nor disagree** | **Disagree** | **Strongly disagree** |
| --- | --- | --- | --- | --- | --- |
| **A1** It is worth fighting for justice. |  |  |  |  |  |
| **EEC5** I feel a lot of anger, rage or aggression. |  |  |  |  |  |
| **RF4** I cannot forgive the people who did not help me. |  |  |  |  |  |
| **IE3** Some of the experiences that I have had were a lot worse than what humans generally experience. |  |  |  |  |  |
| **A3** People need to hear my story. |  |  |  |  |  |
| **RF3** I do not want to forgive, I want revenge. |  |  |  |  |  |
| **A5** I am hopeful that the people, who are responsible for the acts of injustice, will be legally held accountable. |  |  |  |  |  |
| **EEC6** I feel guilty and/or ashamed. |  |  |  |  |  |
| **IP3** I often get less than I deserve. |  |  |  |  |  |
| **EEC7** I feel humiliated. |  |  |  |  |  |
| **IP4** I am suffering because of someone else’s negligence. |  |  |  |  |  |
| **IP5** It all seems so unfair. |  |  |  |  |  |
| **EEC2** I feel unsafe. |  |  |  |  |  |
| **Please choose for each statement to what extent you agree with it.** | **Strongly agree** | **Agree** | **Neither agree nor disagree** | **Disagree** | **Strongly disagree** |
| **IE2** I feel that acts of injustice that I have experienced affected me in a permanent way. |  |  |  |  |  |
| **EEC1** I am scared that I will experience injustice again in my life. |  |  |  |  |  |
| **IP2** My life is a lot harder than the lives of people around me. |  |  |  |  |  |
| **IE1** Some of the experiences that I have made were wrong and acts of injustice. |  |  |  |  |  |
| **RF1** Nothing will ever make up for what I have gone through. |  |  |  |  |  |
| **A2** If another person treats me in an unjust or wrong way, I can successfully protest against it. |  |  |  |  |  |
| **IP1** I am mistreated more often than other people. |  |  |  |  |  |
| **EEC3** I feel betrayed by humanity and cannot trust many people. |  |  |  |  |  |
| **A4** Other people’s compassion helps me when dealing with injustice. |  |  |  |  |  |
| **EEC4** I feel left alone. |  |  |  |  |  |
| **RF2** I want to punish the person who has hurt me. |  |  |  |  |  |

**Appendix B**

**Ukrainian translation of the Perceived Injustice Questionnaire (PIQ)**

| **Будь ласка, виберіть для кожного з тверджень, наскільки ви погоджуєтеся з ним.** | **повністю згоден** | **згоден** | **ані згоден, ані не згоден** | **не згоден** | **зовсім не згоден** |
| --- | --- | --- | --- | --- | --- |
| **A1** Варто боротися за справедливість. |  |  |  |  |  |
| **EEC5** Я відчуваю багато гніву, злості та агресії. |  |  |  |  |  |
| **RF4** Я не можу пробачити людей, які мені не допомогли. |  |  |  |  |  |
| **IE3** Деякі з переживань, які я мав, були набагато гіршими за тi, що люди переживають взагалі. |  |  |  |  |  |
| **A3** Люди повинні почути мою історію. |  |  |  |  |  |
| **RF3** Я не хочу прощати, я хочу помсти. |  |  |  |  |  |
| **A5** Сподіваюся, що люди, які винні у вчиненні актів несправедливості, будуть притягнуті до юридичної відповідальності. |  |  |  |  |  |
| **EEC6** Я відчуваю провину та/або сором. |  |  |  |  |  |
| **IP3** Я часто отримую менше, ніж заслуговую. |  |  |  |  |  |
| **EEC7** Я відчуваю себе приниженим. |  |  |  |  |  |
| **IP4** Я страждаю через чужу недбалість. |  |  |  |  |  |
| **IP5** Все здається таким несправедливим. |  |  |  |  |  |
| **EEC2** Я почуваюся невпевнено. |  |  |  |  |  |
| **Будь ласка, виберіть для кожного з тверджень, наскільки ви погоджуєтеся з ним.** | **повністю згоден** | **згоден** | **ані згоден, ані не згоден** | **не згоден** | **зовсім не згоден** |
| **IE2** Я відчуваю, що акти несправедливості, які я пережив, вплинули на мене назавжди. |  |  |  |  |  |
| **EEC1** Я боюся, що знову зіткнуся з несправедливістю у своєму житті. |  |  |  |  |  |
| **IP2** Моє життя набагато важче, ніж життя оточуючих мене людей. |  |  |  |  |  |
| **IE1** Деякі з моїх переживань були неправильними і стосувалися актiв несправедливості. |  |  |  |  |  |
| **RF1** Ніщо та ніколи не компенсує те, що я пережив. |  |  |  |  |  |
| **A2** Якщо людина поводиться зі мною несправедливо або неправильно, я можу цьому успішно протистояти. |  |  |  |  |  |
| **IP1** До мене ставляться погано частіше, ніж до інших людей. |  |  |  |  |  |
| **EEC3** Я почуваю себе зрадженим людством і не можу довіряти багатьом людям. |  |  |  |  |  |
| **A4** Співчуття інших людей допомагає мені, коли я стикаюся з несправедливістю. |  |  |  |  |  |
| **EEC4** Я відчуваю себе покинутим. |  |  |  |  |  |
| **RF2** Я хочу покарати людину, яка завдала мені шкоди. |  |  |  |  |  |
